# Pharmacokinetic Profiles of Pentoxifylline and Its 5-Hydroxyhexyl Metabolite Administered by Different Doses in Goats †

**DOI:** 10.3390/ani16101524

**Published:** 2026-05-15

**Authors:** Orhan Corum, Tulay Avci, Mustafa Hitit, Pedro Marin, Mario Giorgi, Duygu Durna Corum, Devran Coskun, Serafettin Kartal, Hatice Rumeysa Ceyhan, Fatma Akin, Ibrahim Buyuktaskapulu, Kamil Uney

**Affiliations:** 1 Department of Pharmacology and Toxicology, Faculty of Veterinary Medicine, University of Hatay Mustafa Kemal, Hatay 31060, Türkiye; duygu.durnacorum@mku.edu.tr; 2 Faculty of Veterinary Medicine, University of Selcuk, Konya 42031, Türkiye; tulay.avci@selcuk.edu.tr; 3 College of Agriculture, Food and Natural Resources, Prairie View University, Prairie View, TX 77446, USA; 4 Department of Pharmacology, Faculty of Veterinary Medicine, University of Murcia, 30100 Murcia, Spain; pmarin@um.es; 5 Department of Veterinary Sciences, University of Pisa, San Piero a Grado, 56121 Pisa, Italy; mario.giorgi@unipi.it; 6 Department of Pharmacology and Toxicology, Faculty of Veterinary Medicine, University of Siirt, Siirt 56100, Türkiye; devran.coskun@siirt.edu.tr (D.C.); fatma.akin@siirt.edu.tr (F.A.); 7 Department of Veterinary Medicine, Samandag Vocational School, Hatay Mustafa Kemal University, Hatay 31800, Türkiye; serafettin.kartal@mku.edu.tr; 8 Department of Veterinary Medicine, Health Services Vocational School, University of Osmaniye Korkut Ata, Osmaniye 80100, Türkiye; hrumeysaceyhan@osmaniye.edu.tr; 9 Department of Pharmacology and Toxicology, Faculty of Veterinary Medicine, University of Selcuk, Konya 42031, Türkiye; ibrahimtk84@gmail.com (I.B.); kuney@selcuk.edu.tr (K.U.)

**Keywords:** increasing dose, goats, 5-hydroxyhexyl metabolite, pentoxifylline, HPLC, pharmacokinetics

## Abstract

This study examined dose-dependent alterations in the pharmacokinetics of pentoxifylline (PTX) and its 5-hydroxyhexyl (M-I) metabolite in goats. Goats were administered PTX intravenously at doses of 10, 20, and 40 mg/kg. PTX and M-I plasma concentrations were quantified by HPLC. The t_1/2ʎz_ values of PTX and its M-I showed significant differences between dose groups. The 40 mg/kg dose of PTX showed lower Cl_T_ and V_dss_ values compared to the 10 and 20 mg/kg doses. For PTX and M-I, higher dose-normalized AUC values were obtained at a dose of 40 mg/kg compared to other doses. Compared to other doses, high dose-normalized C_0.08_ was obtained for PTX at a dose of 40 mg/kg, and a low dose-normalized C_max_ was obtained for M-I at a dose of 10 mg/kg. The AUC_0-lastM-I_/AUC_0-lastPTX_ ratios were 8.9%, 9.6%, and 10.6% for dosages of 10, 20, and 40 mg/kg, respectively, with the maximum ratio observed at the highest dosage. The findings indicated that the pharmacokinetics of PTX and M-I at a dosage of 40 mg/kg exhibited considerable variability in goats. This information may be helpful in adjusting the dosage regimen of PTX in goats, but further studies are needed to determine dose-dependent therapeutic efficacy and safety.

## Simple Summary

Pentoxifylline (PTX) is used to treat circulatory and inflammatory conditions, but its pharmacokinetics in goats remains unknown. This study evaluated intravenous PTX and its M-I metabolite at 10, 20, and 40 mg/kg doses. While 10 and 20 mg/kg doses behaved proportionally, 40 mg/kg caused disproportionately higher plasma concentrations and slower elimination for both compounds. Furthermore, M-I consistently achieved much higher plasma concentrations than PTX. Clinically, 10 mg/kg of PTX was safe; higher doses triggered temporary rapid heart rate and salivation. Although these pharmacokinetic variations might not proportionally alter therapeutic outcomes, they provide essential guidance for veterinarians to safely adjust dosages and avoid toxicity.

## 1. Introduction

Pentoxifylline (PTX), 1-(5-oxohexyl)-3,7-dimethylxanthine, is a methylxanthine derivative used in the treatment of peripheral vascular diseases. PTX increases intracellular cyclic adenosine monophosphate and cyclic guanosine monophosphate by non-selectively inhibiting the phosphodiesterase enzymes [1,2,3]. Furthermore, this medication promotes prostacyclin production while inhibiting thromboxane synthesis [1]. PTX enhances the flexibility of red blood cells by elevating erythrocyte ATP and cyclic nucleotide concentrations. This effect decreases blood viscosity by lowering erythrocyte aggregation and increasing fibrinolysis, resulting in lower plasma fibrinogen concentrations [4]. These effects increase blood flow to peripheral tissues and improve their oxygenation, exhibiting hemorheological effects [1]. PTX also has anti-inflammatory, antioxidant, and immunomodulatory effects [1,2,4]. PTX undergoes extensive metabolism in humans and animals, yielding many metabolites, with the principal metabolite being 5-hydroxyhexyl metabolite (M-I, lisofylline), which is formed in erythrocytes and the liver [1,5]. The pharmacological activity of the M-I metabolite is comparable to that of the parent substance and is effective in the treatment of sepsis, cancer, and type 1 diabetes [6].

PTX is approved by the U.S. Food and Drug Administration for the symptomatic management of intermittent claudication linked to chronic occlusive peripheral vascular diseases of the lower limbs [1]. It is also used to treat diabetic ulcers, cancer, endotoxemia, sepsis, vasculitis, seizure disorders, and collagen diseases because of its pharmacological properties [4,7]. PTX is also employed extra-label in veterinary medicine for conditions such as laminitis, cutaneous vasculitis, endometritis–placentitis, and sepsis in horses [8] as well as for contact allergy, atopic dermatitis, vasculitis, and systemic lupus erythematosus in dogs [9,10]. In goats, PTX has been shown to prevent fetal brain damage by reducing intrauterine inflammation [11] and to modulate changes in WBC and TNF-alpha caused by endotoxemia [12].

Pharmacokinetic studies allow for the quantitative determination of the absorption, distribution, metabolism, and excretion processes of drugs and make significant contributions to determining the appropriate dosage regimen for the target type of drug and reducing the risk of residues in food-producing animals [13,14]. The pharmacokinetics of PTX have been established in sheep [15], cattle [16], dogs [17], horses [8,18], chickens [19], rabbits [20], rats [21], and mice [22,23]. Although PTX has been used in some experimental trials in goats, there is no information on its pharmacokinetics [11,12,24]. PTX can be used for the treatment of diseases in goats that are associated with peripheral circulatory disorders and inflammation as a result of its pharmacological properties, but pharmacokinetic studies are needed for its effective use. The pharmacokinetics of PTX have shown significant variation across animal species and doses [8,15,16]; therefore, determination of pharmacokinetic data and dose-dependent pharmacokinetic variation in goats is necessary. This investigation was designed to investigate the pharmacokinetic changes in PTX and its M-I metabolite as a result of the intravenous (IV) administration of escalating doses (10, 20, and 40 mg/kg) of PTX to goats.

## 2. Materials and Methods

### 2.1. Chemicals

PTX (purity ≥ 99%) and M-I metabolite (purity ≥ 99%) analytical standards were purchased from TCI (Tokyo Chemical Industry, Tokyo, Japan) and from Sigma–Aldrich (St. Louis, MO, USA), respectively. HPLC-grade methanol was obtained from Chemlab (Zedelgem, Belgium). Sodium acetate buffer and acetic acid were all of analytically pure and purchased from Merck (Darmstadt, Germany).

### 2.2. Animals

In this study, nine female hair-breed goats weighing 26 ± 2 kg and 15–19 months of age were used. The goats were sourced from a private commercial enterprise located in Sirvan/Siirt. Following the anamnesis and clinical examination findings, the animals were assessed as healthy. For 15 days prior to the experiment, the goats were maintained in ideal hygienic conditions and subjected to regular monitoring to ensure the absence of any residual drug substances. The goats were fed a ration adequate for their developmental stage, and water and hay were provided ad libitum. The animals were maintained under identical circumstances before and during the investigation and were identified using ear tags and numbered collars. The Local Ethics Committee for Animal Research Studies at Siirt University (Siirt, Türkiye) accepted all study protocols (2023/05/29).

### 2.3. Experimental Design

The trial was carried out utilizing a crossover pharmacokinetic strategy, with a 15-day medication washout time between treatments. Nine goats were randomly divided into three subgroups, A, B, and C, with three goats in each group, and the study was conducted using a three-stage process. In the first stage, PTX was administered to group A at a dose of 10 mg/kg, to group B at 20 mg/kg, and to group C at 40 mg/kg. In the second and third stages, groups A, B, and C were rotated to receive different dose groups, and by the end of the study, all animals had received all three dose levels. PTX, dissolved in sterile water at a concentration of 50 mg/mL, was administered into the left jugular vein via a catheter (21G, 0.8 × 38 mm) over 1 min. Blood samples (1 mL) were collected via catheter from the right jugular vein at 0, 0.08, 0.17, 0.25, 0.33, 0.42, 0.5, 0.75, 1, 1.5, 2, 3, 4, 5, 6, 7, 8, 10, and 12 h and placed in lithium heparin-containing tubes. The tubes were gently agitated multiple times and centrifuged (4000× *g* for 10 min) within one hour. The plasma samples were placed into storage microcentrifuge tubes and refrigerated at −80 °C until analysis. In addition, the goats were monitored for adverse effects throughout the study period.

### 2.4. Determination of Plasma Drug Concentrations

Concentrations of PTX and its M-I metabolite were quantified from HPLC (Shimadzu, Tokyo, Japan) with ultraviolet detection, according to the method previously reported [15]. The HPLC system consisted of a UV detector, an autosampler, a pump, a column oven, and a degasser. The eluate was observed using UV light detection with a wavelength of 275 nm. A mobile phase composed of a mixture of sodium acetate buffer (0.025 M) and methanol (60:40, *v*/*v*), and an InertSustain C18 column (4.6 × 250 mm; 5 μm) placed in an oven at 40 °C were used. The flow rate was 1 mL/min and the run time 15 min.

Two hundred microliters of goat plasma aliquot were combined with three hundred microliters of methanol. Following a 45 s shaking period, the samples were subjected to centrifugation at 10,000× *g* for 10 min. Subsequently, the supernatant was transferred to an autosampler vial, and 20 μL was injected into the system. Limits of detection and quantification were 0.02 and 0.04 µg/mL for both PTX and M-I metabolite. The recovery ratio for PTX and M-I metabolite was >93%. The coefficient of variation and bias values for PTX and M-I metabolite were less than 7.44% and less than 6.40%, respectively.

### 2.5. Pharmacokinetic Analysis

A computer application (WinNonlin 6.1.0.173, Pharsight Corp. Mountain View, CA, USA) was utilized to ascertain pharmacokinetic parameters. Non-compartmental analysis was used to determine pharmacokinetic parameters, which are defined and abbreviated in the footnote to Table 1. For PTX, t_1/2ʎz_, MRT, V_dss_, Cl_T_, AUC, AUC_extrap%_, and C_0.08_ values were determined. The t_1/2ʎz_, MRT, AUC, AUC_extrap%_, C_max_, and T_max_ values were calculated for M-I metabolite. The plasma C_0.08_, C_max_ and T_max_ were calculated by direct observation of concentration–time curves. The conversion ratio of PTX to M-I metabolite in different dose groups was calculated with the following formula (AUC_0-∞ M-I_/AUC_0-∞PTX_).

### 2.6. Statistical Analysis

The pharmacokinetic parameters were presented as geometric mean (min–max), except for T_max_, which was given as median. The statistical analysis was conducted using SPSS 22.0, with a *p*-value of 0.05 being statistically significant. The Shapiro–Wilk test was employed to evaluate data normality, while the Levene test was used to check homogeneity. Prior to statistical analysis, AUC, C_max_ and C_0.08_ were standardized to a dosage of 10 mg/kg. The one-way analysis of variance (ANOVA) and post hoc Tukey test were utilized to evaluate differences in pharmacokinetic parameters.

## 3. Results

### 3.1. Safety

No systemic or local side effects were noted in goats after IV injection PTX at doses of 10 mg/kg. However, short-term (approximately 1 h) tachycardia and excessive salivation were observed at doses of 20 and 40 mg/kg. These signs and restlessness were more pronounced at the 40 mg/kg dose.

### 3.2. Pharmacokinetic Parameters of Pentoxifylline

**Figure 1:**
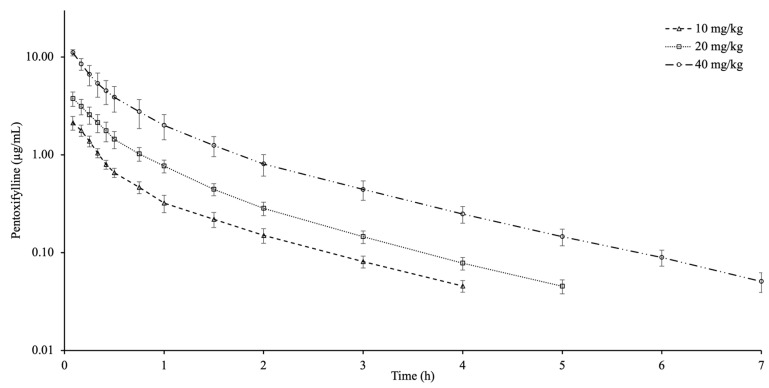
Semi-logarithmic plasma concentration–time curves of pentoxifylline after intravenous administration at doses of 10, 20 and 40 mg/kg in goats (*n* = 9, mean ± SD).

Plasma concentration curves of PTX after injection of different doses in goats are shown in Figure 1. The detection times of PTX in blood varied between dose groups and were detected for up to 4 h at 10 mg/kg, up to 5 h at 20 mg/kg, and up to 7 h at 40 mg/kg. Table 1 shows the pharmacokinetic data in goats after administering different doses of PTX. The t_1/2ʎz_ exhibited notable variations among dose groups. The 40 mg/kg exhibited a higher dose-normalized AUC and C_0.08_, as well as a lower Cl_T_ and V_dss_, in comparison to the 10 and 20 mg/kg. The AUC_extrap_ remained below 6.09% across all dosage groups.

### 3.3. Pharmacokinetic Parameters of M-I Metabolite

**Figure 2:**
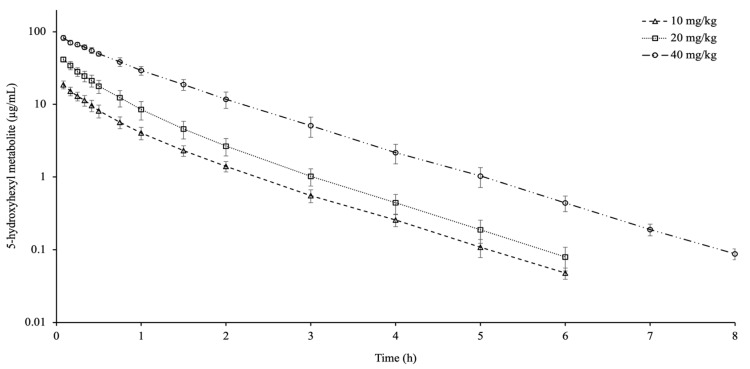
Semi-logarithmic plasma concentration–time curves of 5-hydroxyhexyl metabolite after intravenous administration of pentoxifylline at doses of 10, 20, and 40 mg/kg in goats (*n* = 9, mean ± SD).

Pharmacokinetic data and plasma concentrations of the M-I metabolite following IV injection of different doses of PTX in goats are shown in Table 2 and Figure 2, respectively. M-I metabolite was observed for up to 6 h at doses of 10 and 20 mg/kg and up to 8 h at a dose of 40 mg/kg. The t_1/2ʎz_ showed significant differences among dose groups. The dose-normalized AUC was higher at 40 mg/kg compared to other doses, while the dose-normalized C_max_ was lower at 10 mg/kg compared to other doses. The AUC_0-lastM-I_/AUC_0-lastPTX_ ratio was higher in the 40 mg/kg dose than in the other doses. T_max_ was comparable throughout the dosage groups (*p* > 0.05). The AUC_extrap_ was below 0.76% in all dose groups.

## 4. Discussion

Despite PTX being utilized in several experimental research studies involving goats [11,12], there is a lack of knowledge concerning its pharmacokinetics. This study explains, for the first time, the pharmacokinetics and dose-dependent alterations in pharmacokinetics of PTX in goats. The study concluded that the pharmacokinetics of PTX and its M-I metabolite in goats showed significant differences, particularly at the 40 mg/kg dose.

In goats, IV injection of PTX at a dosage of 10 mg/kg had no adverse effects, but doses of 20 and 40 mg/kg resulted in short-term tachycardia and excessive salivation. The administration of the medication to goats by infusion (0.3 mg/kg/min for 30 min or 0.5 mg/kg/min for 15 min) induced temporary tachycardia and a decrease in body temperature [12,25]. Hematological and biochemical parameters were also altered in goats at concentrations of 10–40 mg/kg [24]. While PTX does not cause any clinical signs in dogs [17,26], it has been documented to cause transient tachycardia, excessive salivation, and restlessness in sheep (IV, 40 mg/kg) [15]; transient excessive salivation and restlessness in cattle (IV, 10 mg/kg) [16]; and tachycardia, sweating, and muscle spasms in horses (IV, 8.5 mg/kg) [18]. Therefore, these adverse effects should be considered when PTX is used in goats, especially at doses of 20 and 40 mg/kg.

In goats, PTX was used as an IV infusion at a dose of 7.5–9 mg/kg (0.3 mg/kg/min over 30 min or 0.5 mg/kg/min over 15 min) and orally at a dose of 30–60 mg/kg [11,12,25]. Hematological and biochemical parameters were altered in goats at concentrations of 10–40 mg/kg; however, it was clinically well tolerated [24]. Although PTX showed some positive effects at a dose of 7.5 mg/kg in endotoxemic goats, it was insufficient to correct the acute phase response [25]. The impact of PTX is dose-dependent, and in rats and mice, elevated dosages have mitigated the adverse effects associated with LPS [17,25]. Therefore, goats were administered PTX at doses of 10, 20, and 40 mg/kg. PTX is recommended to be administered orally or intravenously. In this study, IV administration was preferred to determine dose-dependent changes in Cl_T_ and V_dss_ without the influence of the absorption process [27].

The high V_dss_ of PTX in goats after IV administration at doses of 10–40 mg/kg was 5.82–7.34 L/kg, which was comparable to the value in cattle (6.30 L/kg) [16] and higher than that reported in sheep (0.55–0.66 L/kg) [15], dogs (1.01–4.1 L/kg) [17,26], and horses (1.15–2.81 L/kg) [8,18]. The volume of distribution can vary depending on the physicochemical properties of the drugs, their binding ratio to plasma proteins, and body composition [28]. Although PTX is a hydrophilic drug that is highly soluble in water [29], it generally has a large volume of distribution. Despite its hydrophilic nature, its large distribution volume might be attributed to the plasma protein binding ratio. The data on PTX’s binding ratio to plasma proteins is inconsistent. Some studies report a plasma protein binding ratio of 70% [4], while others report no binding at all to plasma proteins and instead bind to erythrocytes [30]. The differences in PTX’s V_dss_ in animals may be due to variations in body components and plasma/erythrocytes protein binding ratios between species. The V_dss_ value at the 40 mg/kg dose decreased from 7.34 L/kg to 5.82 L/kg in comparison to the 10 and 20 mg/kg doses. However, in sheep, an increase in V_dss_ has been reported in the high-dose group [15]. PTX binds to 45% of erythrocytes, increasing their flexibility and allowing them to penetrate deeper into tissues [31]. The decrease in V_dss_ in goats at a dose of 40 mg/kg may be due to the reduction in Cl_T_ and increase in plasma concentration as a result of saturation of PTX binding to erythrocytes. A drug with a V_dss_ value significantly higher than 0.6 L/kg is considered to dissolve in a volume greater than the total amount of water in the body [32]. In rats and dogs, PTX concentrations in the brain, heart, liver, lungs, kidneys, and skeletal muscle were similar to those in plasma, indicating good tissue distribution [33]. Therefore, the decrease in V_dss_ at a dose of 40 mg/kg may be considered clinically insignificant.

PTX’s Cl_T_ decreased in hepatic impairment but remained constant in renal impairment [31,34]. Cimetidine reduced the Cl_T_ of PTX by inhibiting hepatic microsomal enzyme activity [35,36]. Therefore, the elimination of PTX is more dependent on biotransformation than renal excretion. The Cl_T_ of PTX is 2–4 times higher than that from the hepatic bloodstream, indicating that it is metabolized outside the liver [37]. PTX is metabolized into seven phase I metabolites (I-VII) in humans, with the M-I metabolite being predominant, formed in erythrocytes and liver [4,18]. PTX and M-I are converted into other metabolites in the liver, and less than 1% of both are excreted unchanged in the urine [34]. However, three metabolites of PTX were detected in dog plasma, and six metabolites were detected in horse urine [17]. This shows that the biotransformation of PTX varies among animal species. The high Cl_T_ of PTX in goats after IV injection at doses of 10–40 mg/kg was 5.16–7.21 L/h/kg, which was similar to the value in cattle (5.31 L/kg) [16] and higher than that reported in sheep (0.42–0.64 L/kg) [15], dogs (1.68–2.22 L/kg) [17,26], and horses (2.38–3.06 L/kg) [8,18]. Phase I reactions, such as oxidation and reduction, are important in the metabolism of PTX [4,8,35]. Enzymatic activities associated with biotransformation differ between animal species [38]. This may explain the variation in the Cl_T_ value of PTX across different animal species. The Cl_T_ of PTX in goats decreased after IV injection at a dose of 40 mg/kg in comparison to other doses. Similarly, dose-dependent decreases in Cl_T_ of PTX have been reported in sheep [15]. The dose-dependent decrease in Cl_T_ may be due to saturation of biotransformation enzymes in the liver or erythrocytes, which can lead to reduced metabolism and clearance of the drug at higher doses.

The t_1/2ʎz_ after IV injection of PTX at different doses was 0.90–1.15 h, similar to that previously reported in cattle (1.05 h) [16], sheep (0.68–0.96 h) [15], and chickens (1.05 h) [19], but longer than that reported in horses (0.38 h) [8]. However, the t_1/2ʎz_ value exhibited nearly a tenfold range in dogs, ranging from 0.28 h to 2.73 h [17,26]. Although it has been reported that the t_1/2ʎz_ value is prolonged in sheep in a dose-dependent manner [15], the dose-dependent change in goats was obtained in the following order: 40 mg/kg > 10 mg/kg > 20 mg/kg. The t_1/2ʎz_ is a hybrid pharmacokinetic parameter that is not a direct measure of an independent physiological process and is affected by changes in Cl_T_ and V_d_ values [39], species- and dose-dependent variations may result from differences in these parameters.

M-I is the active metabolite of PTX and has a pharmacological effect similar to the parent drug [6]. Dose-dependent alterations in M-I metabolite pharmacokinetic properties were observed in goats after administration of PTX at various doses. These alterations were most evident at the PTX dosage of 40 mg/kg. After administering PTX to goats at a dose of 40 mg/kg, the AUC value of the M-1 metabolite increased. Similarly, the AUC value of the M-1 metabolite increased in sheep at a dose of 40 mg/kg [15]. The M-I metabolite is converted into other metabolites in the liver, such as PTX, and less than 1% is excreted unchanged in the urine [17]. In this study, the AUC value of the M-I metabolite may have been elevated as a result of metabolic saturation.

The AUC_0-lastM-I_/AUC_0-lastPTX_ ratio was 8.87, 9.62, and 10.62 at the 10, 20, and 40 mg/kg doses administered intravenously, respectively. This conversion ratio was higher than previously reported in cattle (1.34) [16], sheep (0.38–0.46) [15], horses (1.13–2.4) [8], dogs (0.63) [26], and chickens (1.52) [19]. PTX is metabolized to its M-I metabolite in erythrocytes and the liver, and this process is reversible [19,26]. PTX is rapidly metabolized to M-1, resulting in a quick increase in M-1 concentration and a corresponding decrease in PTX concentration within minutes [18]. The peak concentration of the M-I metabolite was achieved at 5 min in this study. Aldo-keto reductase and CYP2E1 enzymes play a key role in this conversion [15,40], and differences in the AUC_0-lastM-I_/AUC_0-lastPTX_ conversion ratio among animal species may stem from differences in the activity of these enzymes [38]. The AUC_0-lastM-I_/AUC_0-lastPTX_ conversion ratio was seen to rise at a dose of 40 mg/kg compared to doses of 10 and 20 mg/kg. However, the conversion ratio in sheep did not exhibit any dose-dependent variations [15]. PTX and M-I metabolism is reversible, and both undergo biotransformation reactions in the liver to be converted into other metabolites. In goats, the conversion rate may have been increased due to dose-dependent changes in various biotransformation processes.

There are no data available on the effective plasma concentrations of PTX in goats. The therapeutic concentration of PTX in humans ranges from 0.5 to 2 μg/mL depending on the indication [41] and >1 μg/mL for TNF-α inhibition in horses [42]. The concentration of PTX required to reduce blood viscosity by increasing deformability is 0.03 μg/mL for stiffened red blood cells and 20 μg/mL for normal red blood cells [22]. Following IV administration of PTX at doses of 10, 20, and 40 mg/kg to goats, the C_0.08_ values were 2.11, 3.73, and 11.07 μg/mL, respectively. These results show that in goats, PTX reached therapeutic concentrations at all three dose levels, excluding normal red blood cell deformability. However, PTX is converted into M-I and M-V metabolites with similar pharmacological effects, and this conversion differs between species, making it difficult to determine its therapeutic effect [34]. In goats, the C_max_ of M-I after IV administration of PTX at dosages of 10, 20, and 40 mg/kg were 18.06, 41.47, and 82.22 μg/mL, respectively. These concentrations were significantly greater than those achieved for PTX. However, since these assessments are based on data reported in other animal species, they may not be applicable to goats, and it is crucial to determine the therapeutic concentrations of PTX and its M-I metabolite under different clinical conditions in goats.

This study has several limitations that impact the applicability of its findings. Although the biotransformation of PTX differs between species, the failure to examine metabolites apart from the M-I metabolite is a deficiency of this study. Although the target site of PTX is erythrocytes, the lack of determination of its binding ratio to erythrocytes and plasma proteins constitutes a deficiency. Furthermore, the lack of investigation into the therapeutic efficacy of PTX and its M-I metabolite is a limitation of the study.

## 5. Conclusions

This study provides the first comprehensive evaluation of the pharmacokinetics of PTX and its active M-I metabolite following IV administration in goats. Although significant pharmacokinetic variability was observed among the 10, 20, and 40 mg/kg dosages—most notably a reduction in Cl_T_ and V_dss_ at the highest dose—these variations do not strictly confirm non-linear kinetics, and their direct clinical relevance remains to be fully elucidated. Importantly, while PTX achieved potentially therapeutic plasma concentrations across all evaluated dose levels, the concentrations of the M-I metabolite were substantially higher, underscoring its pivotal role in the drug’s overall pharmacological profile in this species. From a clinical safety perspective, the 10 mg/kg dose was well-tolerated without adverse events; however, practitioners should exercise caution with higher doses (20 and 40 mg/kg), which precipitated transient adverse effects including tachycardia, excessive salivation, and restlessness. Ultimately, notable pharmacokinetic differences exist among the evaluated doses. However, these variations may not necessarily result in proportional changes to actual therapeutic outcomes (e.g., anti-inflammatory or hemorheological efficacy). Therefore, further clinical investigations are essential to determine the precise therapeutic efficacy, safety margins, and optimal dosage regimens for PTX and its M-I metabolite across various caprine pathological conditions.

## Figures and Tables

**Table 1:** Pharmacokinetic parameters of pentoxifylline following intravenous administrations at doses of 10, 20, and 40 mg/kg in goats (*n* = 9).

Parameters	10 mg/kg	20 mg/kg	40 mg/kg
t_1/2ʎz_ (h)	1.01 (0.91–1.10)	0.90 (0.83–0.96) ^a^	1.15 (1.02–1.27) ^b,c^
AUC_0-last_ (h*µg/mL)	1.35 (1.14–1.62)	2.72 (2.23–3.49)	7.67 (6.29–10.78) ^b,c^
AUC_0-last_/dose (h*µg/mL)	0.13 (0.11–0.16)	0.14 (0.11–0.17)	0.19 (0.16–0.27) ^b,c^
AUC_0–∞_ (h*µg/mL)	1.42 (1.22–1.70)	2.78 (2.28–3.57)	7.75 (6.36–10.90) ^b,c^
AUC_extrap%_ (%)	4.64 (3.38–6.09)	2.08 (1.68–2.36)	1.07 (0.76–1.59)
MRT_0–∞_ (h)	1.04 (0.96–1.11)	1.02 (0.93–1.10)	1.13 (1.06–1.20) ^b,c^
Cl_T_ (L/h/kg)	7.06 (5.85–8.19)	7.21 (5.60–8.74)	5.16 (3.67–6.13) ^b,c^
V_dss_ (L/kg)	7.34 (5.81–9.11)	7.33 (5.86–9.67)	5.82 (4.38–6.93) ^b,c^
C _0.08_ (µg/mL)	2.11 (1.81–2.72)	3.73 (3.15–4.67)	11.07 (10.14–12.54) ^b,c^

^a^: This shows the difference on the same line between 20 mg/kg and 10 mg/kg (*p* < 0.05). ^b^: This shows the difference on the same line between 40 mg/kg and 10 mg/kg (*p* < 0.05). ^c^: This shows the difference on the same line between 40 mg/kg and 20 mg/kg (*p* < 0.05). t_1/2ʎz_: elimination half-life; AUC: area under the concentration–time curve; AUC_extrap%_: area under the plasma concentration–time curve extrapolated from tlast to ∞ in % of the total AUC; MRT: mean residence time; Cl_T_: total body clearance; V_dss_: volume of distribution at steady state; C_0.08_: PTX concentration at the time of initial sampling.

**Table 2:** Pharmacokinetic parameters of 5-hydroxyhexyl metabolite following intravenous administrations of pentoxifylline at doses of 10, 20, and 40 mg/kg in goats (*n* = 9).

Parameters	10 mg/kg	20 mg/kg	40 mg/kg
t_1/2ʎz_ (h)	0.79 (0.70–0.86)	0.74 (0.66–0.80) ^a^	0.84 (0.80–0.88) ^b,c^
AUC_0-last_ (h*µg/mL)	12.51 (9.92–16.18)	26.61 (17.27–33.22)	82.21 (67.21–93.67) ^b,c^
AUC_0-last_/dose (h*µg/mL)	1.25 (0.99–1.62)	1.33 (0.86–1.66)	2.06 (1.74–2.37) ^b,c^
AUC_0–∞_ (h*µg/mL)	12.56 (9.96–16.24)	26.69 (17.32–33.36)	82.32 (67.29–94.94) ^b,c^
AUC_extrap%_ (%)	0.46 (0.32–0.77)	0.28 (0.18–0.51)	0.12 (0.11–0.16)
MRT_0–∞_ (h)	0.95 (0.92–1.07)	0.84 (0.77–0.96) ^a^	1.12 (1.00–1.22) ^b,c^
C_max_ (µg/mL)	18.06 (15.87–21.83)	41.47 (34.89–45.82) ^a^	82.22 (72.26–95.88) ^b^
T_max_ (h)	0.083	0.083	0.083
AUC_0-lastM-I_/AUC_0-lastPTX_ (%)	8.87 (8.15–9.51)	9.62 (7.57–10.59)	10.62 (8.70–12.02) ^b,c^

^a^: This shows the difference on the same line between 20 mg/kg and 10 mg/kg (*p* < 0.05). ^b^: This shows the difference on the same line between 40 mg/kg and 10 mg/kg (*p* < 0.05). ^c^: This shows the difference on the same line between 40 mg/kg and 20 mg/kg (*p* < 0.05). t_1/2ʎz_: elimination half-life; AUC: area under the concentration–time curve; AUC_extrap%_: area under the plasma concentration–time curve extrapolated from tlast to ∞ in % of the total AUC; MRT: mean residence time; C_max_: peak plasma concentration; T_max_: time to reach the peak plasma concentration.

## Data Availability

The data presented in this study are available upon request from the corresponding authors.
